# Recent Advances in Diagnostic Approaches for Epstein–Barr Virus

**DOI:** 10.3390/pathogens9030226

**Published:** 2020-03-18

**Authors:** Mai Abdel Haleem Abusalah, Siew Hua Gan, Mohammad A. I. Al-Hatamleh, Ahmad Adebayo Irekeola, Rafidah Hanim Shueb, Chan Yean Yean

**Affiliations:** 1 Department of Medical Microbiology and Parasitology, School of Medical Sciences, Universiti Sains Malaysia, Kelantan 16150, Malaysia; maiabdelhaleem@student.usm.my (M.A.H.A.); irekeola@student.usm.my (A.A.I.); hanimkk@usm.my (R.H.S.); 2 School of Pharmacy, Monash University Malaysia, Selangor 47500, Malaysia; gan.siewhua@monash.edu; 3 Department of Immunology, School of Medical Sciences, Universiti Sains Malaysia, Kelantan 16150, Malaysia; alhatamleh@student.usm.my; 4 Microbiology Unit, Department of Biological Sciences, College of Natural and Applied Sciences, Summit University Offa, Offa PMB 4412, Kwara State, Nigeria

**Keywords:** Epstein–Barr virus, laboratory diagnostic techniques, carcinoma, exosome

## Abstract

Epstein–Barr virus (EBV) is the causative agent of many diseases including infectious mononucleosis (IM), and it is associated with different subtypes of lymphoma, sarcoma and carcinoma such as Hodgkin’s lymphoma, non-Hodgkin’s lymphoma, nasopharyngeal carcinoma, and gastric carcinoma. With the advent of improved laboratory tests for EBV, a timelier and accurate diagnosis could be made to aid better prognosis and effective treatment. For histopathological lesions, the in situ hybridization (ISH) of EBV-encoded RNA (EBER) in biopsy tissues remains the gold standard for detecting EBV. Methods such as the heterophile antibody test, immunofluorescence assays, enzyme immunoassays, Western blot, and polymerase chain reaction (PCR) are also employed in the detection of EBV in different types of samples. The determination of EBV viral load using PCR, however, is gaining more prominence in the diagnosis of EBV-associated diseases. Given the challenge of false positive/negative results that are sometimes experienced during the detection of EBV, variability in results from different laboratories, and the impact of factors such as sample type and the immunological status of patients from whom samples are collected, the need to critically examine these present methods is invaluable. This review thus presents current advances in the detection of EBV, detailing the advantages and disadvantages of the various techniques. In addition, fundamental virological concepts are highlighted to enhance the greater understanding, the proper application, and the interpretation of EBV tests.

## 1. Introduction

Epstein–Barr virus (EBV) is a member of the Herpesviridae family and is a ubiquitous pathogen that is persistently harbored by people throughout the world. The viral genome is about 170 kb and comprises a linear double stranded DNA molecule that encodes >85 genes. It is encased within a capsid which is surrounded by the viral envelope [1,2]. EBV is found in approximately 95% of the total population. Primary infection with EBV is more frequent during childhood and causes a mild disease. The disease is typically asymptomatic in 20%–80% of individuals by the age of two-to-three years [1,3]. When uninfected teenagers and young adults are exposed to EBV, approximately 30%–70% will develop infectious mononucleosis (IM) [3]. 

EBV can infect a wide range of cells and tissues including T and B lymphocytes, nasopharynx and oropharynx squamous epithelial cells, salivary and stomach glands, thyroid glandular epithelial cells, smooth muscle, and follicular dendritic cells [4]. However, EBV primarily infects and replicates in the stratified squamous epithelium of the oropharynx, followed by a latent infection of B lymphocytes [4]. It has been suggested that the EBV infection of B lymphocytes occurs in the oropharyngeal lymphoid organs [2]. In normal carriers, the virus persists in circulating memory B cells and initiates the production of immunoglobulins [1,2]. Following EBV’s infection of B cells, a specific set of latency-related genes and transcripts are expressed, and the virus could remain dormant in resting memory B cells, from which it intermittently reactivates at any mucosal site where B cells are present (Table 1) [4,5]. The reactivation of EBV poses a great and difficult challenge to infected hosts [3]. In healthy adults, it is estimated that for every million B cells in circulation, approximately 1 to 50 are infected with EBV, with the number of latently-infected cells in each individual remaining stable for several years [6]. Therefore, EBV coexists with most human hosts without obvious outcomes. However, in some people, the virus is associated with the development of certain malignancies [2].

The EBV infection of B lymphocytes results in two outcomes with respect to the physiological impacts of antigen stimulation. The first outcome leads to the production of memory B cells that persist for a long period, which is subsequently associated with dormant viral persistent. Meanwhile, the second outcome results in the differentiation of B cells into plasma cells that are programmed to die [3,7]. This results in lytic replication, which is accompanied by the expression of several viral proteins, including the trans-activator protein BZLF1 (otherwise called ZEBRA) and viral protein complexes that are collectively known as early antigen (EA) and viral capsid antigen (VCA), leading to the elicitation of the humoral immune response [4,8]. In the course of the lytic cycle, regulatory proteins such as immediately early antigen (IEA) and EA groups are sensitized to permit the production of viral DNA (EBV-DNA), VCA and membrane proteins (MAs) [9]. 

Furthermore, an in vitro study demonstrated that from the approximately 100 viral genes that are expressed during replication, only ten are expressed in latently-infected B cells [10]. There are different types of RNA and proteins expressed in the latently-infected B cells. They include non-coding RNAs (EBV-encoded small RNA 1 (EBER1) and EBER2, small non-coding RNAs, microRNAs, EBV-stable intronic-sequence RNAs (EBV-sisRNAs), EBV small nucleolar RNAs (EBV-snoRNAs) and RPMS1 messenger RNA), six nuclear proteins (Epstein–Barr virus nuclear antigen 1 (EBNA1), EBNA2, EBNA3A, EBNA3B, EBNA3C, and EBNA5) and two latent membrane proteins (latent membrane protein 1 (LMP1) and LMP2) [5,11]. The diverse expression programs of EBV-encoded proteins apparently rely on the type, differentiation, and activation status of the infected B cells [10]. During the latency phase, viral proteins are reduced to evade the recognition of infected cells by cytotoxic T cells [4,5,10].

It has been suggested that tumor necrosis factor receptors (TNFRs) might be induced by LMP1 with a higher expression of TNFR–associated factor 1 (TRAF1) observed in EBV-associated carcinomas [12]. There is increasing evidence to suggest that a high TNFR2 expression (as preferentially expressed on cancer cells and immunosuppressive cells) in a cancer microenvironment impacts cancer progression, metastasis, and immune evasion [13,14]. Additionally, circular RNAs (circRNAs), a unique class of conserved non-coding RNA, have been found to play a vital role in EBV latency and the reactivation of B lymphocytes and epithelial cells into several EBV-associated carcinomas [11,15]. As such, circRNAs have potential future use as therapeutic targets, prognostic biomarkers, and diagnostic biomarkers for EBV-associated diseases. Therefore, as many evidences have suggested, EBV may play a critical role in the development of different types of diseases and cancers that could be linked to its characteristic latency phase in lymphocyte cells. Hence, this article highlights the involvement of EBV in several diseases with detailed discussion on the most common techniques utilized in the detection of the virus.

## 2. EBV-Associated Diseases

EBV was first discovered through its relationship with African Burkitt lymphoma. It is a causative agent for IM (commonly known as kissing disease) and has also been detected in oral hairy leukoplakia [16]. Previous reports have shown that particular latent EBV-transcription programs are exhibited in numerous human tumors, including immunoblastic lymphoma in immunosuppressed patients, Burkitt lymphoma, Hodgkin’s lymphoma, and nasopharyngeal carcinoma (NPC) [2,4,17,18]. These typical expression patterns act as rough guidelines to aid in the clinicopathological diagnosis of every type of EBV [4,17]. The investigation of patients with EBV-infected tumors has provided a reasonable degree of proof that EBV was present before neoplastic transformation, which highlights the need to further elucidate how much EBV contributes to tumorigenesis [4]. EBV is also associated with autoimmune diseases, including rheumatoid arthritis, Sjogren’s syndrome, systemic lupus erythematosus, and multiple sclerosis [19,20,21]. Furthermore, the virus is associated with a wide variety of benign and neoplastic diseases including posttransplant lymphoproliferative disorder (PTLD) and NPC (which are almost exclusively EBV-related), Hodgkin’s and non-Hodgkin’s lymphomas, and gastric carcinoma (Table 2). On the other hand, many other types of sarcoma are less consistently EBV-related [22].

## 3. Diagnoses of EBV-Associated Diseases

The physical presence of EBV inside a given neoplasm suggests that it may be implicated in the pathogenesis of clonal expansion in EBV-associated diseases [4]. As such, EBV can be used as a biomarker to diagnose and assess tumor spread as well as to monitor treatment. For this reason, the laboratory testing of EBV and the identification of viral gene products have become essential because EBV is considered a helpful tumor marker [25]. Currently, there are several diagnostic methods for EBV detection, including serological and molecular diagnostic methods, although each has their own limitations (Table 3). 

Despite the fact that in situ hybridization (ISH) is the gold standard method for detecting EBV-associated carcinoma with a sensitivity of 100%, the molecular determination of viral DNA, RNA and EBV viral load is currently being utilized in the clinical assessment of tumor-associated EBV infections [25,31]. While viral culture may be used as an alternative semi-quantitative method, it is not preferable in clinical laboratories due to its high cost, slow turnaround time, and the need for trained personnel [4]. However, accurate laboratory tests to detect EBV are important in fundamental and epidemiological research. From a clinical perspective, tests for EBV will help to determine correct diagnoses for patients [32]. Moreover, with various diagnostic methods available, the detection of EBV also aids during treatment monitoring and the prognosis of EBV-associated diseases [31,32].

### 3.1. EBER-ISH

EBER-ISH is deemed the gold standard for the detection of latent EBV in tissue samples [4]. It has been shown that EBER transcripts are expressed not only in tumor cells infected with EBV but also in lymphoid tissues taken from IM patients [33]. EBER (which consists of non-polyadenylated and non-coding RNAs) represents two major viral RNA transcripts, EBER1 (166 nucleotides) and EBER2 (172 nucleotides), in latently-infected cells [31,34,35]. These small RNAs are localized to the nucleus, but they could also localize in the cytoplasm since they can bind to diverse nuclear and cytoplasmic proteins [35]. They act as inhibitors to interferon-mediated antiviral activity and apoptosis [25,32]. Because viral genome copy numbers are usually low during the latent phase because significant parts of the genome are silent, procedures that target viral DNA or messenger RNA (mRNA) have been difficult to apply, particularly to clinical samples [33,36,37]. In contrast, EBERs are abundantly expressed (million copies) in latently-infected cells. Hence, they are useful markers for detecting latent EBV infection in tissue samples [36,38]. 

ISH utilizes EBERs as a standard method in detecting and confirming EBV in tumor biopsy samples [25]. However, in suspected IM, clinical findings and serology are usually sufficient for diagnosis, while, due to the histological interference between non-Hodgkin’s and Hodgkin’s lymphoma, it is possible to get counterproductive [25]. EBER ISH can be performed using DNA or RNA probes, such as riboprobes and peptide nucleic acid probes, on paraffin or cytological samples [4,32]. However, commercially-available EBER tests may be labeled with biotin, digoxigenin, or fluorescein [4,39]. The paraffin segment DNA-ISH that utilizes radiolabeled probes has many disadvantages including being a tedious, time-consuming method with a high possibility of obtaining false negative results [40]. 

Due to RNA degradation, false-negative EBER hybridization using ISH is possible [32]. Thus, control hybridization must be run in parallel to ensure that RNA is protected and is obtainable for probe binding [4,32]. The U6 (non-coding small nuclear RNA) cellular transcript, which has similar size, intranuclear distribution, and abundance to EBER, is considered a suitable control for ISH [32]. Despite this, the precise interpretation of results depends on the ability of the pathologist to recognize tumor cells from background lymphocytes or artifacts [4]. In conclusion, the incorporation of other diagnostic methods in addition to EBER-ISH may increase the diagnostic accuracy of different EBV-associated diseases and the subsequent monitoring of effective treatment.

### 3.2. EBV Serology

The immune system is a crucial first-line defense against viral infections. In immunocompetent patients, natural killer (NK) cells and cytotoxic T lymphocytes are involved in controlling the development of transformed cells [41,42,43]. However, the response is deficient and cannot ensure the total destruction of the virus, allowing the virus to maintain a low or periodic viral production for life [9,41]. Following recuperation, it has been demonstrated that one out of 10,000–100,000 memory B cells contains episomal EBV-DNA [43]. Additionally, in immunocompetent patients, the latent cells are regularly eliminated by cytotoxic T lymphocytes, while in immunosuppressed patients, the transformed cells cause different lymphoproliferative disorders [43]. Interestingly, antibodies (Abs) against antigens in both the lytic and latent stages are involved in the humoral response. However, only a few have generally been investigated and utilized for diagnostic purposes [9]. It has been shown that anti-EA antibodies (EA immunoglobulin G (IgG)) are of two forms; EA-diffuse (D) IgG, which increases within three-to-four weeks and could remain until three-to-four months, and EA-restricted (R) IgG, which may remain at a high level for up to two years [9,44]. In some cases, EA (D) IgG is detectable several years after a primary infection. High titers of EA (D) IgG, VCA and EA IgA have been observed in patients with NPC [44,45,46]. 

In certain cases of protracted disease, EA (R) IgG remains detectable following the waning of EA (D) IgG, as seen in patients below two years old, Burkitt lymphoma patients, and recently infected patients. Both types of EA, (D) and (R), have also been observed in immunocompromised patients and in cases of reactivation [44,45,47]. Though antibodies against VCA IgG and immunoglobulin M (IgM) commonly appear at the time when patients show clinical symptoms of acute infection, IgG remains positive throughout the patients’ lives, while IgM disappears within a few weeks, though the levels may persist for several months [48]. However, some patients with primary infection are negative for VCA IgM [49]. Meanwhile, anti-EBNA-2 IgG (EBNA-2 IgG) appears early, while anti-EBNA-1 IgG (EBNA-1 IgG) is typically not detectable during the initial three-to-four weeks following clinical symptoms and is therefore an indication of past infection [49,50,51]. In addition, EBNA-1 IgG is mostly negative in immunosuppressed patients and in patients with persistent infection, while IgM-VCA Abs appear early during infection and normally disappear within four-to-six weeks [46,49,52]. However, IgG-VCA Abs appear in the acute stage and remain positive for life [53]. Therefore, in immunocompetent patients, VCA IgG, VCA IgM, and EBNA-1 IgG are the most utilized markers to differentiate acute from past infection [54]. 

The types and levels of antibodies among immunocompromised individuals may change with the dynamics of the disease, in which atypical profiles may be detected [40,49]. Therefore, in these cases, the detection of antibodies may not be decisive. Similarly, in EBV-associated tumors, patients may have high titers of VCA IgG and EA IgG, as well as a low titer of EBNA-1 IgG [55]. Nevertheless, the characteristic profile of NPC is high levels of both VCA and EA IgA, which may suggest the disease’s site of origin (nasopharyngeal mucous membrane) [56,57]. Because there are 32 possible serological patterns of antibodies against EBV that can be generated, there is a high possibility of misinterpretation, and it remains a challenge for physicians to identify [49,51,58,59]. However, in addition to patients’ follow-up to evaluate any changes in the antibody profile (as some cases may have distinctive profiles), it is also helpful to perform other laboratory tests as an additional precaution, although this may incur additional costs [49]. Various methods are available for the serological detection of EBV antibodies, each with their own advantages and limitations. Some of these methods are discussed below.

#### 3.2.1. Heterophile Antibody Test

The heterophile antibody (HAb) test (informally called the “monospot test”) was first introduced in 1932 [29]. It is the most utilized serological test and is considered a simple but nonspecific test. HAb, which is usually used in the diagnosis and screening of both primary and recurrent infections, is considered as a sensitive diagnostic test for IM [25,29,48]. The HAb test is dependent on the ability of a patient’s serum or plasma to agglutinate horse, goat, or sheep erythrocytes [29,33,48]. Normally, antibodies are detected in high concentrations during IM but not during numerous other diseases [60]. However, the test is frequently supplanted by latex agglutination tests, which are modern alternatives that detect the serum-mediated agglutination of latex beads covered by bovine heterophile antigens (monospot assays), and enzyme-linked immunosorbent assays (ELISAs) [9,25,29,48].

It has been estimated that during the course of EBV infection, 85%–90% of adults and teenagers are positive, with approximately 50% tested positive for the HAb in the first week [60]. However, the detection rate when using this test is much lower in children. Only approximately 10%–30% of children less than two years old and 50% of children between two and five years old are positive [60,61]. Apart from high false negative results in children, the HAb test has other disadvantages including being nonspecific such that it could generate false positive results in non-EBV infections (such as viral hepatitis, rubella, malaria, and HIV), malignancies, and autoimmune disorders [48,49,51,61] Additionally, because the disease may be latent for one year or more, the test does not generally imply an acute EBV infection [48,61]. 

#### 3.2.2. Specific EBV Antibodies Tests

Specific EBV antibodies tests are tedious, time consuming, and costlier than monospot tests [29]. In addition, these tests utilize distinctive substrates or antigens. There are distinctive types of specific tests for anti-EBV antibodies (VCA IgG, EBNA-1 IgG, IgM, and EA IgG) [29]. Normally, the routine EBV diagnosis includes three methods: 1) immunofluorescence assays (IFAs), 2) enzyme immunoassays (EIAs), such as luminescence-based detection and solid-phase enzyme linked immunosorbent assay (ELISA); and 3) a Western blot assay that is performed with another test such as chemiluminescence immunoassay (CLIA) variants for confirmation [9,49]. In addition, newer multiplex flow immunoassays (MFIs) are also used [62]. 

##### IFA and EIA

The IFA is considered a gold standard and often utilizes EBV-transformed Burkitt lymphoma cell lines (e.g., the P3HR-1 or Raji cell lines), although their sensitivity is similar to that of EIAs [9]. However, EIAs are primary EBV-specific methods that utilize the synthetic peptides or fusion proteins, as well as purified native or recombinant proteins (represent either the total VCA-encoded gene or just segments of the VCA-encoded gene). An EIA can be run in an automated format, thus, enabling the investigation of a large number of samples [49]. The EBNA-1 EIA can be fabricated to be more sensitive than the IFA (as far as there is the prior identification of anti-EBNA-1 antibodies), whereas IFAs are equivalent or less sensitive than the VCA EIA assay (for IgG and IgM) [9,49]. Nevertheless, some supplementary diagnostic tests that can help characterize acute infections or other phases of infection are required. These tests include the Western blot and avidity test for specific IgG antibodies [63]. 

##### Western Blot

To detect specific EBV antibodies using specific EBV antigens simultaneously, Western blot analysis incorporates several methods, including line blot assays with recombinant antigens, such as EBNA-1 (p72), VCA (p18 and p23), EA (p54 and p138), and MAs (gp 350/250), and traditional lysate blot tests (with EBV-transformed cells). Meanwhile, the latest line blot assay utilizes IEA (ZEBRA) [9,49]. However, the VCA antigen p18 is believed to be a substitute marker in the absence of EBNA-1 IgG because anti-p18 IgG is mostly produced late in the course of the disease, thus allowing for the detection of EBV-specific antibodies to different EBV-specific antigens [49]. In addition, anti-p18 IgG is present in the case of immunosuppression, making stage-specific diagnostic tests convenient to replicate and thus justifying the utilization of the test as a confirmatory method [9,49]. Furthermore, when differentiating acute from chronic infections in cases that are VCA IgG-positive but EBNA-1 IgG- and VCA IgM-negative, using immunoblotting is particularly valuable. Likewise, immunoblotting is utilized in patients with acute infection to identify VCA IgM but not against p72 IgM [9]. Nonetheless, a lack of standardization of buffer conditions and the combination of recombinant antigens and the lysates from cell lines are still notable imperfections in immunoblotting analysis [9,49]. However, for the accurate diagnosis of EBV-associated diseases, some supplementary diagnostic tests, such as the avidity test, can perhaps be run concurrently.

#### 3.2.3. Avidity Testing

Avidity provides information on the strength of antibody binding to multivalent antigens. Avidity is estimated following a short incubation of the antigen-antibody complex with urea [63]. High-avidity antibodies enable binding to the antigen, while low-avidity antibodies are eliminated following the addition of urea [63,64]. VCA IgG avidity testing can distinguish primary from past infections in anti-EBNA-1 negative cases, as well as in the absence of VCA IgM [49,63,64]. Hence, the analysis of a particular EBV IgG avidity allows for more precise estimates of the exact dates of infection because avidity rises progressively over the course of infection, and, thus, it may be a good alternative to EA IgG testing [54]. Furthermore, the development of IgG in vivo can be estimated in vitro by determining avidity, since B cells are capable of changing from IgM to IgG isotypes in vivo [63]. The first IgG antibodies are considered to have a low avidity, while after somatic hypermutation in IgG antibodies, B-cell clones eventually produce IgG antibodies with a higher avidity [49]. However, the kinetics of IgG development (which may be accomplished within a few weeks following primary EBV infection) may vary in different individuals [49]. Therefore, estimation is performed by a VCA EIA-specific substrate, a Western blot procedure, or an IFA [9]. 

Quantitative immunofluorescence has been utilized in combination with urea to assess avidity. Urea primarily enables the disaggregation of antibody/antigen complexes [64] Usually, test slides are first incubated with serum dilutions. Subsequently, parallel slides are either treated with urea or secondary fluorescent antibodies (which is more common), followed by a washing step and the procedure being parallelly performed for the control [9,49,63,64]. The correlation between the titers is then utilized to determine the avidity index. Nevertheless, the technique normally requires a high standardization of immunofluorescence titer quantitation [63]. Though the developmental kinetics of VCA-IgG avidity is rapid, all sera from acute EBV infections tend to indicate low avidity in the first 10 days following infection [63]. The determination of VCA IgG avidity can assist in the diagnosis of primary EBV infection, particularly for VCA IgM-negative cases, as well as in cases with long-term persistent VCA IgM, which underpins the event of past diseases without EBNA-1 IgG (should the avidity index be high) [49]. Therefore, this methodology is useful because antibody avidity is directed against different specific antigens that are measurable in a single test [63]. However, it has been shown that due to the higher concentration of recombinant antigen in this test, avidity determination is much slower than in immunofluorescence detection [63].

To run the test, it is possible to use individual antigens (e.g., p23, p18, p54, p138, and p72) or a combination of the antigens [9]. Though IgG avidity determination has been shown to be helpful in differentiating acute from past EBV infections (using p23, p18, and p72), EA-IgG avidity determination is not useful because of the high fluctuations observed [63]. However, the limitations of the avidity test include the different development rates of antibodies in individuals, and it cannot be utilized in newborns due to the presence of maternal antibodies [9,49].

### 3.3. Molecular Assays

For patients at risk of EBV-related lymphoproliferative disorders and EBV-related malignancies, the detection of EBV-DNA by molecular techniques is valuable for diagnosis and follow-up treatment [9]. Because the onset of symptoms in IM is often hidden up to the fifth day of disease, there is a paucity of details regarding virus–host interactions. It is estimated that the viral load in the oral cavity (1–2 log10 EBV copies/ml in oral cells) is higher than that in whole blood [48]. Furthermore, viral clearance from blood is much faster than from the oral region, where the viral load may remain high for several months in saliva and oral cells [48]. 

The quantitative measurement of EBV-DNA is necessary for distinguishing between healthy carriers and patients with EBV-related diseases [25]. Various molecular techniques have been established and utilized for the identification of EBV-DNA and to measure viral load [43]. To date, ISH, RNA, and protein-based assays, quantitative real-time polymerase chain reaction (qPCR) and immunoblotting have been utilized in the diagnosis of EBV and in determining the stage of infection [65]. Though these methods help in diagnosis, due to the absence of standardization, the differences in sensitivity and specificity observed among laboratories ought to be systematically considered [9,44,65]. In addition to investigating other serological markers, more recent investigations have demonstrated that qPCR is an imperative technique, especially for the diagnosis of EBV acute infection and silent reactivation, because it is considered sensitive, reliable, stringent, simple, specific, precise, and fast [9]. Furthermore, it is broadly used when monitoring patients with a high risk of developing EBV-related diseases or those with immunocompromised status. However, the threshold at which medical involvement is required, the units of estimation for viral load, and the best samples to be utilized for DNA testing remain unstandardized [44]. 

The qPCR method depends on the amplification of a conserved nucleic acid sequence (ideally ∼100 bp) and utilizes a fluorescent probe or an intercalating dye to quantify the targeted nucleic acid against serial dilutions of known EBV-DNA concentrations [25]. Since the reaction mixtures are contained in sterile, closed vessels, the risk of amplicon contamination is limited [25]. However, there could be variations in the degree of EBV-DNA detections in different laboratories due to differences in diagnostic kits, equipment, and procedures [66]. Numerous types of samples can be tested by qPCR, as shown in Table 4. However, there is much controversy regarding the ideal type of sample (tissue, peripheral blood mononuclear cells (PBMCs), serum, or plasma) that should be utilized to investigate EBV-DNA. Additionally, there is inconsistency in the measurement unit used since they are either reported as copies per 100,000 white blood cells (WBCs), DNA concentration, milliliter, or microgram [9,44,67]. EBV-DNA in confirmed infected patients can be detected within 14 days of symptoms. Though viral load gradually diminishes in PBMCs following the initiation of the immune response, the reduction occurs at a faster rate in plasma or serum and becomes undetectable after three-to-four months [9,43]. However, memory B cells infected with EBV may be present in the latent stage for a long time in the blood [44]. However, it is essential to consider inter-individual variations in EBV kinetics, as the viral load may take up to one year to achieve a steady low level in some individuals, depending on the person’s immune status and condition [44].

It has been estimated that there is 1–50 copies of EBV-DNA/10⁶ WBCs in healthy carrier’s blood, although EBV-DNA is undetectable in serum or plasma [9]. However, the detection of EBV-DNA in the patient’s serum is particularly useful in the early stages of acute infection and is more sensitive than serology or IgG avidity tests [44]. Nevertheless, in immunocompetent patients with acute infection, it is not typical to test for EBV-DNA because serology is regarded as adequate (even in cases with negative or questionable serological results in which there is a strong clinical doubt of infection) [9]. The investigation of EBV-DNA is valuable in immunocompromised patients who show higher viral loads than healthy carriers, particularly in patients with negative or ambiguous serological test findings [9,68]. Additionally, EBV-DNA can be detected in episomes or virions from lysed tumors or latent EBV-infected cells in the blood of patients with EBV-related malignancies [9,69]. Aside from patients with AIDS-related brain tumors due to decreased blood levels resulting from the protection conferred by the blood–brain barrier, detecting EBV-DNA is particularly valuable in patients with EBV-related tumors [9].

In Hodgkin’s lymphoma patients, serum is useful for detecting EBV-DNA as this disease involves the migration of episomal or naked EBV-DNA derived from apoptotic cells to the circulatory system [43]. Similarly, in NPC, cancer cells multiply in the tissue and migrate to the peripheral blood; hence, cell-free EBV-DNA is detectable in the peripheral blood [70]. Conversely, in PTLD, the disease biology includes the migration of blast B lymphocytes into the circulation, thus making the utilization of peripheral blood mononuclear cell samples preferable. Interestingly, viral load relates to disease severity in lymphoproliferative disease and EBV-associated malignancies, making it a useful prognostic marker [44]. Nevertheless, the improper storage of whole blood can cause either a false-positive finding if EBV-DNA leaves the intracellular compartment or a false-negative finding if the nucleases degrade plasma EBV-DNA [21,29,69]. 

### 3.4. Exosomes as Promising Biomarkers

Exosomes are extracellular microvesicles surrounded by a lipid bilayer membrane that is strengthened with macromolecules such as lipids, carbohydrates, proteins, and nucleic acids (microRNA (miRNA), mRNA, and DNA) with size ranging from 30 to 150 nm in diameter and densities of 1.13–1.21 g/mL [71]. These microvesicles are usually released from most eukaryotic cell types, including dendritic cells, epithelial cells, endothelial cells, T cells, B cells, reticulocytes, neurons, and cancerous cells [71,72]. In addition, exosomes are present and can be detected in almost all body biological fluids, such as serum, plasma, semen, breast milk, cerebrospinal fluid, urine, saliva, ascites fluid, amniotic fluid, and bronchoalveolar lavage fluid [71,72]. Currently, exosomes have been reported to have a critical role in cell to cell communication due to a unique lipid bilayer membrane’s exosome components [71,72]. It has been shown that exosomes play important role in the pathogenesis of several viral diseases, such as EBV, herpes simplex virus, HIV-1, hepatitis C and human T-cell lymphotropic virus type 1 (HTLV-1), and in the pathogenesis of different cancers, such as lymphoma, melanoma, glioma, NPC, gastric carcinoma, colorectal cancer, and breast and ovarian cancers [71,73]. 

**Figure 1:**
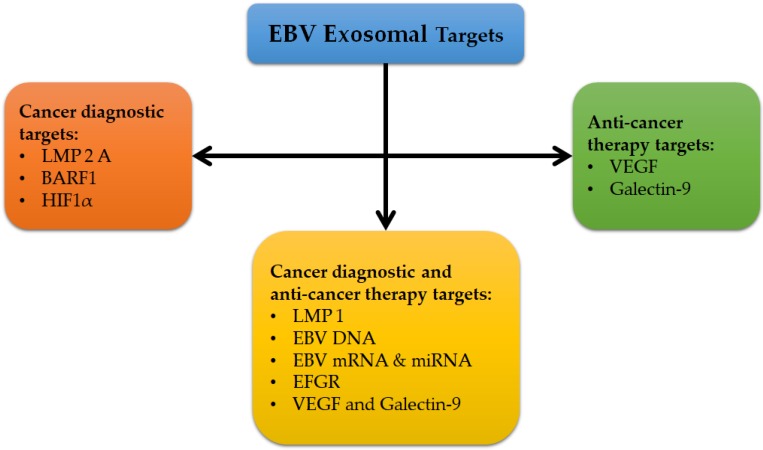
EBV exosomal target protein for cancer diagnostics and anticancer therapies [71,72,73].

Currently, exosomes’ function has been examined in several studies, especially in EBV. They have critical roles in different biological and pathological processes, such as cell proliferation, evasion from the immune system, angiogenesis, carcinogenesis, and metastasis [73]. EBV exosomes have been found to embed different types of viral components such as LMP1, LMP2A, BamHI-A rightward frame 1 (BARF1), and EBV nucleic acid (DNA, mRNA, and miRNA) [71,72,73]. Hence, these EBV exosomes targets can be helpful diagnostic and prognostic markers, as well as anticancer target therapies in different types of EBV-associated carcinomas and diseases (Figure 1). The simultaneous detection of different pathogenic markers on exosomes could be employed as a sensitive and specific diagnostic method, as well as in monitoring treatment response in different EBV-associated carcinomas. The molecular technique of qPCR is an ideal method for this detection, especially in detecting EBV mRNAs and EBV miRNAs, which are more stable than other EBV pathogenic genes and proteins because they are both encapsulated inside the exosomes and protected from ribonuclease (RNase) degradation [71].

Currently, exosome detection has several limitations, including low specificity and sensitivity. This is because the EBV-associated markers that are carried by exosomes are not specific to a particular type of cancer and have low or no detectable expression during the early stage of the disease [71]. In addition, while the most common methods to isolate exosomes are ultracentrifugation and density gradient centrifugation, these methods may not be able to isolate exosomes with high purity [71,72]. Therefore, more reliable and specific isolation methods are needed in the future. While exosomes may have the potential to become specific and sensitive biomarkers for the diagnostic and therapy response prediction of different EBV-associated carcinomas in the future, more investigations are needed to validate the pathogenic EBV exosomes for the clinical diagnosis of EBV-associated carcinoma.

## 4. Conclusion and Future Directions

Testing VCA (IgG and IgM) and EBNA-1 IgG in serum samples is usually sufficient to diagnose IM. In the case of intermediate results, other tests, such as PCR, Western blot, heterophile antibody assay, and avidity testing, are recommended. Though the gold standard for primary EBV infection diagnosis is still the IFA, the EIA is considered a good alternative in terms of sensitivity and specificity. Recently, the method of choice in the diagnosis of EBV-related diseases has been EBV viral load determination by PCR. The diagnostic value of PCR has contributed to the rapid incorporation of this test into routine medical practice as the method of choice in the early diagnosis of different EBV-related diseases, monitoring the efficacy of therapy and prognosis. However, additional information and the better assessment of patient’s condition can be accomplished by testing the expression of EBV-associated genes by using quantitative methods such as qPCR in addition to measuring the EBV load. These quantitative methods will help to understand the pathogenesis of EBV-associated diseases and to manage patients with high viral loads. Currently, EBV-related neoplasia is accurately diagnosed by EBER-ISH (using biopsy samples) and EBV viral load tests (using blood samples).

However, further evidence and consensus are needed to standardize the procedures, such as sample type, preparation, primer/probe designs, equipment, protocols, reporting unit, and intervention threshold. As such, a major goal in future is to standardize these tests for the accurate detection of EBV. It is likely that further innovations, such as proteomics assays and gene expression profiling, will reveal unique patterns of viral and human gene expression corresponding to EBV diagnosis, prognosis, and outcomes of treatment.

## Figures and Tables

**Table 1:** Epstein–Barr virus (EBV) in infected B-cells with EBV latency pattern and associated malignancy.

	Infected Cells				
	Native B-cells	Germinal Center B-cells	Peripheral Memory B-cells	Dividing Peripheral Memory B-cells	Plasma Cells
Transcription program	Latency III	Latency II	Latency 0	Latency I	Lytic
Viral proteins	All EBNAs, EBERs, LMP-1, LMP-2A and LMP-2B	EBNA-1, EBERs, LMP-1 and LMP-2A	EBERs	EBNA-1 and EBERs.	All lytic genes
Function of viral proteins	Activate B-cell	Differentiate activated B-cell into memory B-cell	Allow for lifetime persistence	Allow for the virus in latency-programmed cell to divide	Assist viral replication in plasma cells
Associated malignancies	IM and post-transplant lymphoproliferative disorder	Nasal NK cell lymphoma, Hodgkin’s lymphoma, chronic active EBV infection, NPC and peripheral NK/T cell lymphoma	Healthy carrier	Burkitt lymphoma and gastric carcinoma	IM and NPC
Specimens for measuring viral load	Plasma or serum, MNCs and WBC	Plasma or serum, MNCs (for chronic active EBV infection), tissue biopsy	Plasma or serum, WBC	Plasma or serum	Plasma or serum

EBV, Epstein–Barr virus; EBNA, Epstein–Barr virus nuclear antigen; LMP, latent membrane protein; EBERs, EBV-encoded small RNAs; NK cells, natural killer cells; NK/T cell, nasal natural killer (NK)/T-cell; MNCs, mononuclear cells; WBC, white blood cell; IM, infectious mononucleosis; NPC, nasopharyngeal carcinoma.

**Table 2:** Diseases associated with EBV infection.

Tumor	Subtypes	Association with EBV (% cases)	References
Autoimmune disease	Multiple sclerosis	99	[23]
	Systemic lupus erythematous	99	[23]
	Rheumatoid arthritis	88	[23]
	Sjogren’s syndrome	57	[20]
XLP	XLP1 and XLP2	65	[24]
Benign reactive infection	Infectious mononucleosis	>99	[25]
	Oral hairy leukoplakia	>95	[25]
	Chronic active EBV infection	100	[25]
Nasopharyngeal carcinoma	Non-keratinizing	100	[26]
	Keratinizing	30–100	[26]
Gastric carcinoma	UCNT	100	[26]
	Adenocarcinoma	5–15	[26]
**Non-Hodgkin’s Lymphoma and Related Neoplasms**			
Burkitt lymphoma	Endemic	100	[27]
	Sporadic	10–80	[27]
	AIDS-associated	30–40	[27]
B-lymphoproliferative disease	Post-transplant	>90	[27]
	HIV-related	>90	[27]
Diffuse large B cell lymphoma	NOS	10	[27]
	PAL	100	[27]
	HIV-related	20–60	[27]
Rare immunocompromised B lymphomas	Plasmablastic lymphoma	75–90	[27]
	Primary effusion lymphoma	75–90	[27]
T/NK lymphoproliferative disease	CAEBV	100	[27]
	Extra-nodal T/NK lymphoma	100	[27]
	Aggressive NK lymphoma	100	[27]
**Hodgkin’s Lymphoma**			
NLPHL	-	<4 (usually absent)	[28]
Classical Hodgkin’s lymphoma	All subtypes	40	[29]
	Nodular sclerosis	10–40 (variably present)	[27,30]
	Mixed cellularity	70–80 (usually present)	[27,30]
	Lymphocyte depleted	10–50 (variably present)	[27,30]
	Lymphocyte rich	30–60 (variably present)	[27,30]
	HIV-related	>90	[27,30]

XLP, X-linked lymphoproliferative disease; PAL, pyothorax-associated lymphoma; CAEBV, chronic active EBV infection; NOS, not otherwise specified; NK/T-cell, nasal natural killer/T-cell; HIV, human immunodeficiency virus; AIDS, acquired immune deficiency syndrome; UCNT, undifferentiated carcinomas of nasopharyngeal type; and NLPHL, nodular lymphocyte-predominant Hodgkin’s lymphoma.

**Table 3:** Advantages and disadvantages of various EBV diagnostic methods.

Method	Advantages	Disadvantages
Molecular methods (PCR and other nucleic amplification methods)	(1) Ability to differentiate between healthy carriers and patients with EBV-related disease based on viral load (low or high) (2) Low risk of contamination and reduced labor costs and turnaround time in qPCR (3) Allow for quantitative EBV DNA detection to monitor disease status. (4) Rapid (within 1 to 2 days) (5) More reliable than serological methods in terms of evaluating EBV status in immunocompromised patients (6) For early intervention, it is useful in screening high-risk populations and in monitoring EBV reactivation (7) Sensitive and specific across a wide dynamic range	(1) Could generate false-positive results due to improper blood sample storage and false-negative results due to the presence of nucleases (2) Lack of standardization (3) Expensive (4) Require special equipment
ISH	(1) Ability to identify EBV DNA or EBER transcripts in EBV–associated tumors. (2) Highly reliable confirmatory test for EBV (gold standard for EBV diagnosis)	(1) Only applicable to cells (2) Requires special skills (3) Could get counterproductive due to the histological interference between non-Hodgkin’s and Hodgkin’s lymphoma (4) EBER is downregulated in oral hairy leukoplakia
Heterophile antibody test	(1) Can measure heterophile antibodies released against serum viral proteins (2) Can differentiate between late primary infection and reactivation (3) Cost effective and easy to perform	(1) Less sensitive and less specific (especially in children) (2) Possibility of false-positive result in some cases of autoimmune disease (3) Possibility of false negative is high in young children
IFA (immunofluorescence assay)	(1) Gold standard reference method (2) Highly specific (3) Allows for the staging of EBV infections	(1) A high degree of variability (2) Lacks standardization (3) Equivocal diagnosis of acute EBV infection
EIAs and ELISA	(1) Rapid method (2) More sensitive than the IFA (3) Suitable for automation (4) Inexpensive (5) Less hands-on time	(1) Less specific (2) Difficulty in the staging of EBV infection (single patient’s serum) (3) Lack of standardization (4) Equivocal diagnosis of acute EBV infection
CLIA (chemiluminescence immunoassay)	Sensitive and specific in distinguishing primary infection (transient) from past infection	Requires further validation
Immunoblotting analysis	(1) Highly specific (2) Confirmatory method (3) Possibility of detecting the stage of EBV infection from serum (4) Detection of EBV-specific antibodies against several antigens	(1) Lack of the standardization of buffer conditions, the combination of recombinant antigens and the lysates from cell lines (2) Expensive
Immunoglobulin G (IgG) avidity testing	(1) Confirmatory test for intermediate results (2) Specifies the period of primary infection (3) Distinguishes active from past infections	(1) Depends on the individual maturation rates of antibodies (2) Not useful in newborns (due to maternal antibodies)
Viral cell culture	A precise and semi-quantitative method	(1) Expensive and time consuming (4–8 weeks) (2) Performed only in special laboratories (3) Requires trained personnel

**Table 4:** Prevalence of EBV in various samples (adapted from Smatti MK et al. 2018 [44]).

Country	Sample Type	Sample Size	Seroprevalence (%)	Diagnostic Assay Used	Year
USA	Whole blood	143	42 (29.3)	qPCR	2012
	Whole blood	92	75 (82)	In-house qPCR	2012
	Plasma	116	15 (13)	-	-
	PMNCs	64	56 (88)	-	-
	Oral wash: cell pellet	143	66 (46)	-	-
	Supernatant		61 (42.6)	-	-
	Whole blood	19	5 (26)	qPCR	2016
	Whole blood	66	42 (64)	qPCR	2013
	Whole blood	86	7 (8)	qPCR	2016
Colombia	Saliva	17	9 (52.9)	In-house qPCR	2016
Brazil	Saliva	100	60 (60)	Nested PCR	2018
	Saliva and fresh tissue	17 each	64.7	Nested PCR	2016
	samples		35.3		
	Scraping samples of the tongue lateral border	53	53 (100)	Nested PCR	2008
Australia	Tissue	55	55 (100)	DNA sequence analysis	2012
CzechRepublic	Whole blood	29	19 (66)	qPCR	2011
	Plasma	29	22 (76)		
Poland	Fresh frozen tumor tissue oropharyngeal cancer	78	40 (51.3)	Nested PCR	2016
	Saliva	40 healthy	8 (20)	-	-
	Saliva	56	22 (39.3)	Nested PCR	2004
Sweden	Cervical secretion	305	32 (10.5)	qPCR	-
Germany	Saliva	47	14 (30)	PCR	2017
Serbia	Tissue	80	37 (46.6)	Nested PCR	2016
Qatar	PMNCs	673	354 (52.6)	qPCR	2013
China	PMNCs	859	206 (24)	PCR-RFLP	2017
	Plasma	1318	69 (5.2)	qPCR	2013
	Saliva	20	20 (100)	qPCR	2015
	Paraffin-embedded tissues	209	146 (69.9)	qPCR	2014
India	Serum	40	37 (92.5)	Standard PCDE and PCR	2016
Egypt	Paraffin-embedded samples of breast tissue	84	32 (38)	Nested PCR	2017
Eritrea	Formalin-fixed paraffin-embedded breast cancer tissue	144	40 (27.77)	PCR	2017

PMNCs, peripheral blood mononuclear cells; RFLP, restriction fragment length polymorphism; and PCDE, phenol chloroform DNA extraction.
